# Beyond LIF Neurons on Neuromorphic Hardware

**DOI:** 10.3389/fnins.2022.881598

**Published:** 2022-07-05

**Authors:** Mollie Ward, Oliver Rhodes

**Affiliations:** Department of Computer Science, University of Manchester, Manchester, United Kingdom

**Keywords:** SpiNNaker, dendritic computation, Hodgkin-Huxley, neuronal modeling, neuromorphic computing, spiking neural networks

## Abstract

Neuromorphic systems aim to provide accelerated low-power simulation of Spiking Neural Networks (SNNs), typically featuring simple and efficient neuron models such as the Leaky Integrate-and-Fire (LIF) model. Biologically plausible neuron models developed by neuroscientists are largely ignored in neuromorphic computing due to their increased computational costs. This work bridges this gap through implementation and evaluation of a single compartment Hodgkin-Huxley (HH) neuron and a multi-compartment neuron incorporating dendritic computation on the SpiNNaker, and SpiNNaker2 prototype neuromorphic systems. Numerical accuracy of the model implementations is benchmarked against reference models in the NEURON simulation environment, with excellent agreement achieved by both the fixed- and floating-point SpiNNaker implementations. The computational cost is evaluated in terms of timing measurements profiling neural state updates. While the additional model complexity understandably increases computation times relative to LIF models, it was found a wallclock time increase of only 8× was observed for the HH neuron (11× for the mutlicompartment model), demonstrating the potential of hardware accelerators in the next-generation neuromorphic system to optimize implementation of complex neuron models. The benefits of models directly corresponding to biophysiological data are demonstrated: HH neurons are able to express a range of output behaviors not captured by LIF neurons; and the dendritic compartment provides the first implementation of a spiking multi-compartment neuron model with XOR-solving capabilities on neuromorphic hardware. The work paves the way for inclusion of more biologically representative neuron models in neuromorphic systems, and showcases the benefits of hardware accelerators included in the next-generation SpiNNaker2 architecture.

## 1. Introduction

A vast array of brain modeling techniques exist to simulate brain activity with a view to gaining understanding of the human brain. These techniques range from mathematical representations of individual molecules within neurons to whole-brain simulations. One widely used method for simulation of brain activity is through the use of neural networks. Spiking Neural Networks (SNNs) use biologically-inspired models of neurons to carry out computation with the aim of simulating neural activity and have applications in a number of research areas including computational neuroscience, machine learning, and robotics.

State-of-the-art large scale SNN simulations such as those described by the Blue Brain Project (Markram et al., 2015) aim to mimic brain activity through the use of complex neuron models to advance understanding of the human brain. Scientists were able to accurately reproduce anatomical and physiological features of real biological networks when simulating 0.29 mm^3^ of the rat brain. Despite these recent achievements in the complexity and scale of SNNs simulated, simulation of these SNNs consumes considerable power (megawatts) for simulation of very small regions of the brain (Markram et al., 2015). The full simulation involved over 30,000 different neuron models incorporating 13 different ion-channel models, each neuron comprised on average 20,000 differential equations representing synaptic connections and ion-channels. The full simulation required solving of over two billion equations for every second of biological time (Kumbhar et al., 2019). This power consumption is not required in the human brain which demonstrates a remarkable ability for large amounts of fine-scale computation at a fraction of the power (up to 20 watts) and much faster than SNNs simulated on conventional computer hardware which do not run in real-time (Cox and Dean, 2014).

Energy-efficient neuromorphic systems are designed to mimic the brain and provide low-power platforms for simulation of SNNs, providing a potential solution for the high energy requirements of large scale simulations. As neuromorphic computing platforms target real-time large-scale simulations of SNNs, the biological plausibility of neuron models has been largely ignored in favor of simple, efficient neuron models such as the Leaky-Integrate-and-Fire (LIF) neuron model. Such models are favored due to the ease of solving the equations involved: the differential equations can be solved exactly with a small number of addition and multiplication operations. These simple neurons have allowed large-scale SNNs in real-time such as the cortical microcircuit simulated on SpiNNaker (Rhodes et al., 2020). This SNN simulated ≈1mm^2^ of mamillian neocortex, and while this demonstrated the potential of neuromorphic hardware as a neuroscience research tool, the model does not exhibit the fidelity typically explored by the neuroscience research community.

The LIF model falls short of biological plausibility in two main areas: membrane conductance and structure. Membrane conductance is described with a single term in the model but is actually governed by a number of different ion-channels spanning the neural membrane. The flow of ions through these channels gives biological neurons a wide range of firing capabilities not captured with the LIF model, e.g., the ability to respond to identical inputs differently depending on the current state of the neuron and its ion-channels. Structure is simplified in the LIF model to a single point, however in biology, neurons are complex and elongated and incorporate vast branched extensions called dendrites. Dendrites are active structures capable of generating their own action potentials and are believed to contribute significant computational function to biological neurons (Dayan and Abbott, 2005; Poirazi and Papoutsi, 2020).

Neuron models can increase in complexity to capture these simplified biological features and a wide range of spiking neuron models exist. Hodgkin and Huxley (1952) described a biologically inspired model incorporating equations for sodium and potassium ion channels which govern the progression of the action potential. Other models, such as the Izhikevich model (Izhikevich, 2004), aim to capture certain biological characteristics with more efficient non-biologically plausible equations. However, this lack of biological plausibility takes away the ability to explore the effects of incorporating different ion-channels and more complex morphologies than a single point neuron structure. Accurate and efficient ion-channel modeling on neuromorphic hardware would therefore allow exploration of a wide range of biologically inspired models including multi-compartment models describing complex neural morphologies with dendritic compartments (Markram et al., 2015; Gidon et al., 2020). Implementation of more complex neuron models onto neuromorphic systems could provide low-power solutions for large-scale SNN simulations.

Neuron models with increased complexity have been tested in analog and digital neuromorphic systems, demonstrating the importance of this kind of modeling. For example, individual ion-channels have been modeled in an analog circuit (Abu-Hassan et al., 2019). Here, the aim was to design a biologically accurate neuromorphic circuit that responds identically to a biological neuron under any injected current. The authors were able to reproduce biological voltage recordings with 94–97% accuracy. These neurons were built to demonstrate the potential for making synthetic neurons with therapeutic potential for implementation into the central nervous system, therefore do not easily scale up to large SNNs and do not incorporate structural morphology. However, this work demonstrates accurate representation of ion-channel models on neuromorphic systems. Multi-compartment neuron models have also been tested on neuromorphic systems. BrainScalesS (Schemmel et al., 2010) is an analog neuromorphic system that features an Adaptive-Exponential Integrate-and-Fire (AdExp) neuron model. Schemmel et al. (2017) and more rec ently Müller et al. (2022) and Kaiser et al. (2022) expanded this neuron model to capture dendritic computation in multi-compartment approaches. Intel's Loihi (Davies et al., 2018) also offers support for dendritic computation by offering the opportunity to model neurons with multiple compartments. Here, the additional compartments are effectively identical, the only difference being that the “somatic” compartment generates spike output and the “dendritic” compartments do not. While this does enable a concept of dendritic computation through the ability to distribute synaptic input across individual units, there is a lack of biological plausibility as dendrites are actually much more computationally complex, exhibiting non-linear processing of synaptic inputs (Gidon et al., 2020; Poirazi and Papoutsi, 2020).

### 1.1. Neuromorphic Hardware

While a range of neuromorphic computing systems are currently developed across industry and academia (Schemmel et al., 2010; Benjamin et al., 2014; Furber et al., 2014; Merolla et al., 2014; Davies et al., 2018; Pei et al., 2019), the application of this technology remains limited. While these systems boast impressive performance figures in terms of energy and processing speed, their bespoke architectures are often tailored to particular applications, making it hard to adapt these systems to emerging research problems. The SpiNNaker neuromorphic computing system is selected as the research platform for this work, as its flexibility enables exploration of the target neural models, while constraints such as co-location of memory and processors mean findings remain relevant for the wider neuromorphic research community. The SpiNNaker system is currently an active research platform, with a 1M core machine operating and maintained by the University of Manchester, UK. In parallel to exploring SNN applications on this system, research and development into next-generation hardware is also on-going in the form of the SpiNNaker2 system (Mayr et al., 2019). The two platforms are explored in this work, implementing models on both the SpiNNaker system and a SpiNNaker2 prototype chip (Jib2), to enable comparison and evaluation of performance.

#### 1.1.1. SpiNNaker

SpiNNaker is a massively-parallel many-core digital computing platform, designed for large-scale real-time simulation of SNNs. The system comprises chips assembled into a two-dimensional mesh network, enabling the system to scale to 1M cores. Each individual chip houses 18 cores, network on chip (NoC) and external RAM controller; while each core contains an ARM968, direct memory access controller, communications controller, two timers and other peripherals. Each core has 32kB instruction and 64kB data tightly coupled memory (ITCM and DTCM, respectively), with single cycle access. Each chip has an additional 128MB shared memory, typically accessed *via* DMA, and used to store larger SNN data-structures such as synaptic matrices. Cores operate at 200MHz, running an event-driven operating system enabling efficient neural processing (Rhodes et al., 2018). Individual cores simulate a collection of neurons using software to solve mathematical models representing neural dynamics. These models are solved in discrete time, with the goal of matching the simulation timestep to the time required to process the state update, in order to achieve real-time simulation. Models are programmed in C, and compiled into ARM code using the GCC toolchain. As the core has no floating-point unit, all models are coded using fixed-point arithmetic, with the ISO standard *accum* type favored for the majority of variables. This 32-bit type is a signed representation, with 16 integer and 15 fractional bits, and lower/upper limits of 0.000030517578125 and 65535.999969482421875, respectively (Rhodes et al., 2018). While transcendental functions are also not supported in hardware, division and exponential functions are available in software, requiring approximately 100 clock cycles each. This framework enables real-time implementation of multiple neuron models, including the current- and conductance-based LIF and Izhikevich neurons (Rhodes et al., 2018).

#### 1.1.2. Jib2—SpiNNaker2 Prototype

While the architectural principles are similar, the goals of SpiNNaker2 are to increase the number of cores by a factor of 10, and to increase the number of simulated neurons by a factor of 50, while staying within the same power budget. The system will use an ARM cortex M4 core, with adaptive body biasing to enable increased clock frequencies during periods of high load—switchable from 150 to 300MHz. Additional performance increases are expected from inclusion of hardware accelerators for specific operations common in neural processing, including random number generation, *e*^*x*^, and a single-precision floating point unit (Mikaitis, 2020). The experiments reported in this work are performed on a SpiNNaker2 prototype system known as Jib2, containing 8 processing elements (PE) arranged in two quad processing elements (QPEs) (Höppner et al., 2021). Each PE has an ARM cortex M4 in addition to the above mentioned accelerators, and runs compiled C code in a similar fashion to SpiNNaker (Section 1.1.1), again compiled with the GCC toolchain. PEs each have 128kB of fast access SRAM, for combined instruction and data storage. Jib2 has variable voltage-frequency levels enabling low-power operation and workload-dependent scaling of clock frequency. The experiments reported in this work are performed with the core running with voltage-frequency settings of 0.5V–150MHz and 0.8V–300MHz.

### 1.2. NEURON Simulation Environment

New models implemented on neuromorphic hardware need to be benchmarked again standard methods used in the industry in order to ensure the models are accurate and valid. NEURON is a widely used platform for simulation of individual neuron models and networks of neurons and was designed specifically to simulate equations describing nerve cells. NEURON was chosen as the benchmark for models as it is a standard tool in the research field. It provides an environment for implementing biologically realistic models with a focus on incorporation of multiple ion-channel models and complex branched neuronal morphologies (Hines and Carnevale, 1997). The activity of neurons is modeled using the cable equation in which neurons are treated as trees consisting of a number of compartments. Each compartment is an unbranched cable which can be split into sections and each section can contain its own biophysical properties through different ion-channels. Each section is described by its membrane potential and a set of coupled differential equations are solved for each section within a neuron to compute the evolution of membrane potential inside the neuron over time. The general form of the cable equation for each section, *j* is:


(1) $$\begin{array}{l} c_{j}\frac{dv_{j}}{dt}+I_{m}^{j}=\underset{k}{\sum }\frac{v_{k}-v_{j}}{r_{jk}} \end{array}$$

where *c*_*j*_ is the membrane capacitance of the section, *v*_*j*_ is the membrane voltage of the section, *t* is time, the ionic component $I_{m}^{j}$ includes all currents through ion-channels. $\underset{k}{\sum }\frac{v_{k}-v_{j}}{r_{jk}}$ represents the sum of axial currents entering from neighboring sections, *v*_*k*_ is the membrane voltage of the neighboring section and *r*_*jk*_ is the resistive coupling between compartments. This differential equation is coupled to an additional set of differential equations describing the active states of any ion-channels incorporated into the model. This leads to a set of coupled differential equations which need to be solved at each simulation time step. NEURON uses a backward Euler implicit integration method as standard (Hines and Carnevale, 1997). Each time step update is divided into a set of operations which are performed in order to progress from one time step to the next. These operations include a spike delivery step where synapses are activated by incoming spikes, a matrix assembly step where the ionic and synaptic currents are calculated, a matrix resolution step in which the membrane potential is calculated, a state variable update step in which the ion-channel states are updated, and a threshold detection step in which membrane voltages are checked against threshold values to determine whether a firing condition has been met (Kumbhar et al., 2019). The NEURON platform was designed specifically to model systems of neurons incorporating easy to configure biological data (branched morphologies and ion-channel models) and is therefore widely used by the computational neuroscience community.

## 2. Methods

**Figure 1:**
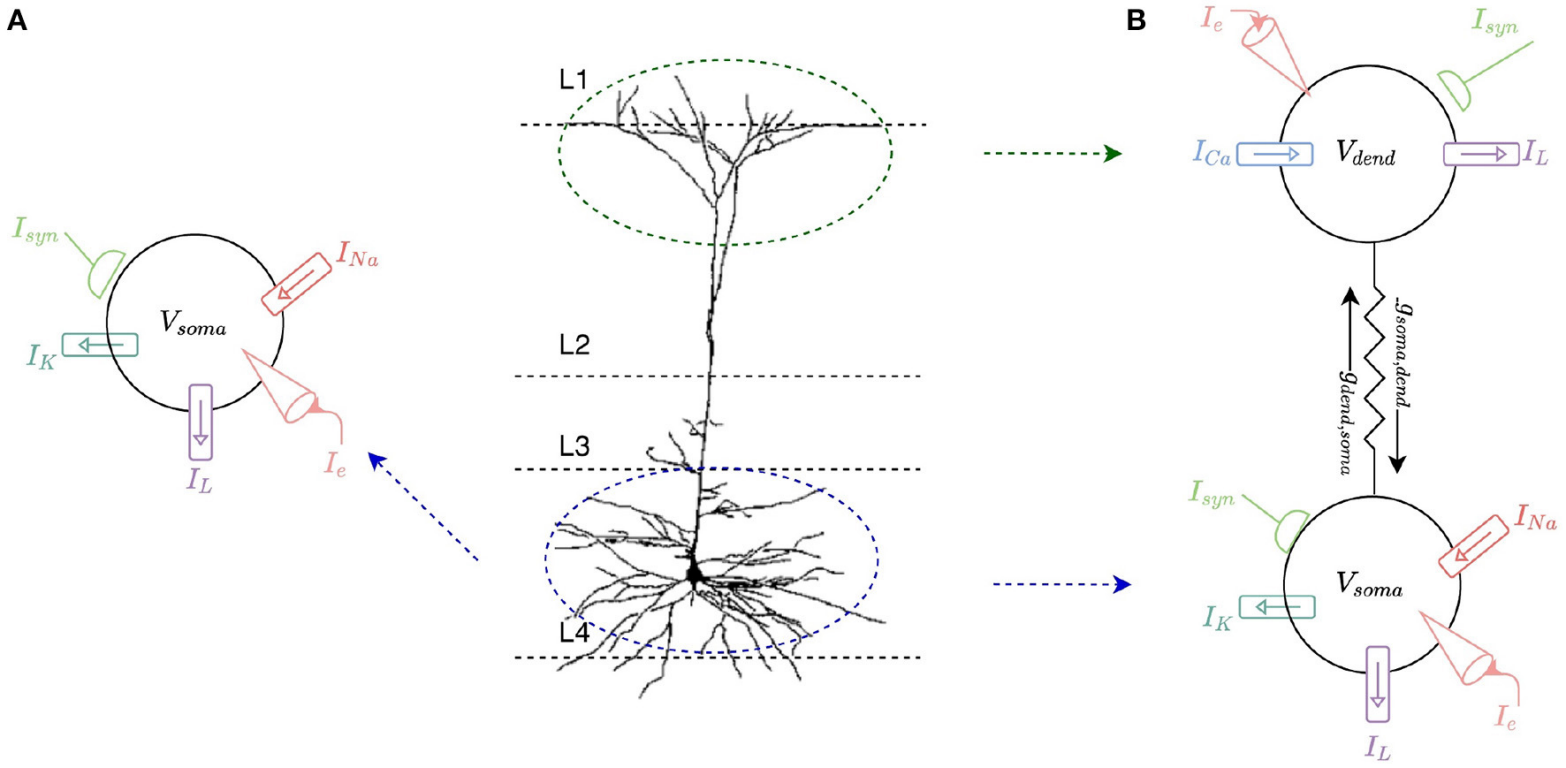
**(A)** Single-compartment HH model of a L2/L3 pyramidal neuron. The soma of the cell is modeled with a leak current, *I*_*L*_, as well as sodium, *I*_*Na*_, and potassium, *I*_*K*_, currents. Current can be injected into the model, *I*_*e*_, and it can receive multiple synaptic inputs, *I*_*syn*_. **(B)** Two-compartment model of an L2/L3 pyramidal neuron consisting of a somatic and a dendritic compartment. The dendritic compartment incorporates a calcium ion channel current, *I*_*Ca*_, and a leak current, *I*_*L*_. The somatic compartment incorporates the same leak current, *I*_*L*_, as well as sodium, *I*_*Na*_, and potassium, *I*_*K*_, currents. The compartments are connected by coupling conductances, *g*_*soma,dend*_ and *g*_*dend,soma*_. Current can be injected into either compartment, *I*_*e*_, and each compartment can receive multiple synaptic inputs, *I*_*syn*_.

This work involves modeling a L2/L3 pyramidal neuron^1^. These neurons comprise approximately two-thirds of neurons in the cerebral cortex of human brains and are key for a large number of cognitive processes, making them prime candidates for mathematical modeling and simulation. Differential equations are used in individual models of spiking neurons to calculate a neuron's membrane potential over time. The change of the membrane potential is proportional to the rate of change of charge build up, i.e., the rate of change of ion flow into and out of the cell, and hence is proportional to the amount of current entering the cell. The amount of current entering the cell is based on the membrane and synaptic conductances and any current injected into the cell. The soma of the neuron is first modeled as a single compartment Hodgkin-Huxley (HH) (1952) model with sodium and potassium ion-channels. This single-compartment model is then expanded to a two-compartment model to capture a dendritic compartment which incorporates a calcium ion-channel model (Figure 1).

### 2.1. Ion-Channels and HH Neurons

A single compartment HH neuron model is built to represent the somatic membrane potential of a typical L2/L3 pyramidal neuron. The model describes the region in which action potentials are generated (Figure 1A). The somatic model incorporates sodium (*I*_*Na*_), potassium (*I*_*K*_), and leak (*I*_*L*_) currents with corresponding maximal conductances *g*_*Na*_ = 0.12 S/cm^2^, *g*_*K*_ = 0.036 S/cm^2^, and *g*_*L*_ = 0.0003 S/cm^2^, and reversal potentials *E*_*Na*_ = +50 mV, *E*_*K*_ = −77 mV and *E*_*L*_ = −54.3 mV. The rate functions for somatic ion channels are modeled as described by Hodgkin and Huxley (1952) and total membrane current in the somatic compartment is calculated as the sum of these three individual currents:


(2) $$\begin{array}{l} I_{soma}=g_{L}\left(V_{soma}-E_{L}\right)+g_{K}n^{4}\left(V_{soma}-E_{K}\right)\\ +g_{Na}m^{3}h\left(V_{soma}-E_{Na}\right) \end{array}$$

where *n*, *m* and *h* are gating variables for the ion channels; *n* and *m* are activation variables for K^+^ and Na^+^ ion channels, respectively, and *h* is an inactivation variable for Na^+^ channels. *n*, *m*, and *h*, like *V*_*soma*_, all vary over time and can be modeled by:


(3) $$\begin{array}{l} \tau_{n}\left(V\right)\frac{dn}{dt}=n_{\infty }\left(V\right)-n \end{array}$$

where


(4) $$\begin{array}{l} \tau_{n}\left(V\right)=\frac{1}{\alpha_{n}\left(V\right)+\beta_{n}\left(V\right)}\text{ and }n_{\infty }\left(V\right)=\frac{\alpha_{n}\left(V\right)}{\alpha_{n}\left(V\right)+\beta_{n}\left(V\right)} \end{array}$$

with similar equations for *m* and *h*. α_*n*_(*V*) and β_*n*_(*V*) are the opening and closing variables for the K^+^ channel, α_*m*_(*V*) and β_*m*_(*V*) are the opening and closing variables for the Na^+^ channel and α_*h*_(*V*) and β_*h*_(*V*) are the key inactivation and de-inactivation variables for the Na^+^ channel. Rate functions for K^+^ and Na^+^ conductances are parameterized according to (Dayan and Abbott, 2005):


(5) $$\begin{array}{l} \alpha_{n}=\frac{0.01\left(V_{soma}+55\right)}{1-\exp\left(-0.1\left(V_{soma}+55\right)\right)}\\ \text{ and }\beta_{n}=0.125\exp\left(-0.0125\left(V_{soma}+65\right)\right) \end{array}$$

(6) $$\begin{array}{l} \alpha_{m}=\frac{0.1\left(V_{soma}+40\right)}{1-\exp\left(-0.1\left(V_{soma}+40\right)\right)}\\ \text{ and }\beta_{m}=4\exp\left(-0.0556\left(V_{soma}+65\right)\right) \end{array}$$

(7) $$\begin{array}{l} \alpha_{h}=0.07\exp\left(-0.05\left(V_{soma}+65\right)\right)\\ \text{ and }\beta_{h}=\frac{1}{1+\exp\left(-0.1\left(V_{soma}+35\right)\right)} \end{array}$$

**Figure 2:**
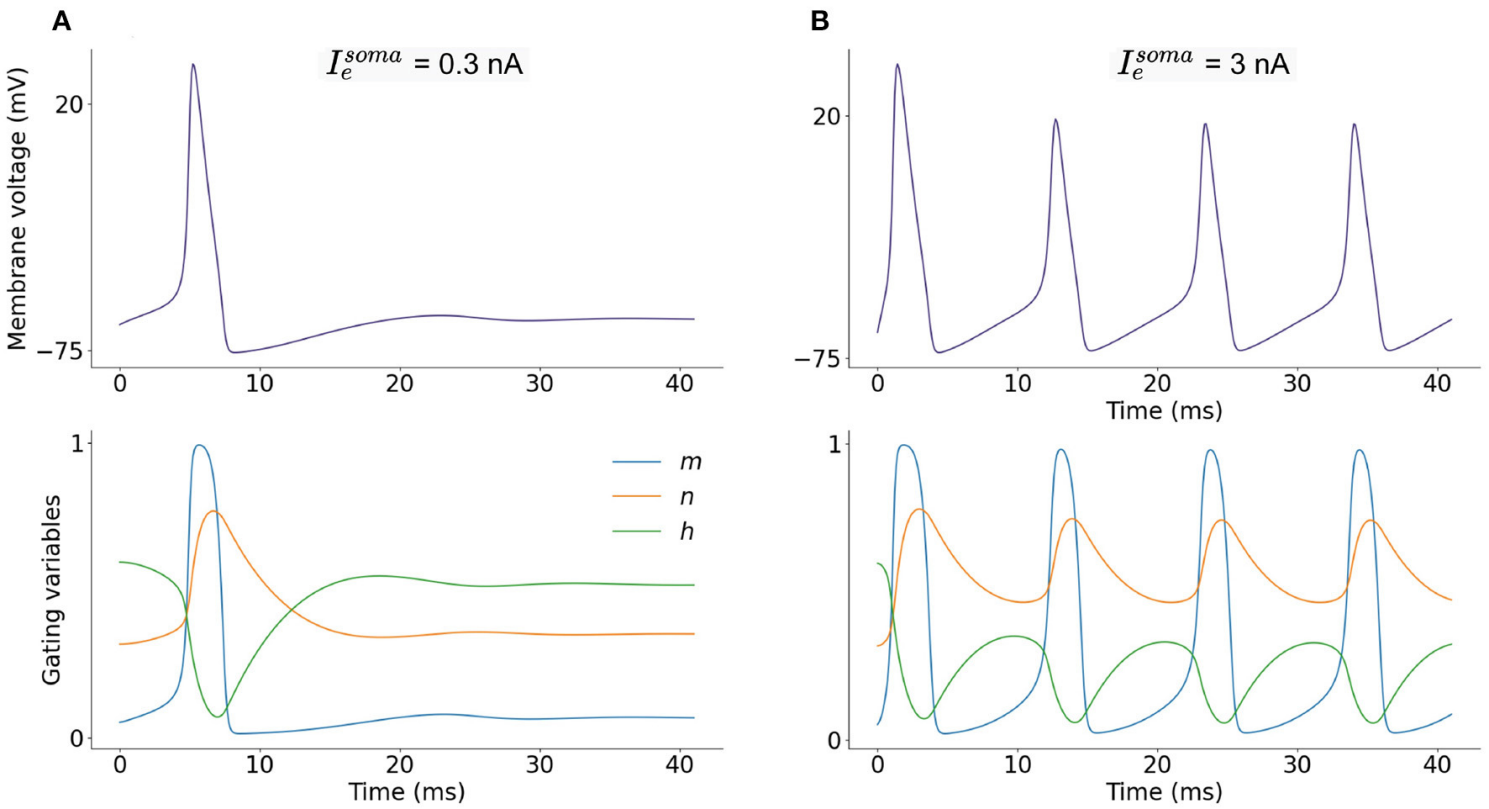
**(A)** Single-compartment model of a L2/L3 pyramidal neuron with injected current $I_{e}^{soma}$ = 0.3 nA and corresponding dimensionless ion-channel activation parameters *m*, *n*, and *h* which govern sodium, *I*_*Na*_, and potassium, *I*_*K*_, currents. **(B)** Current injection of $I_{e}^{soma}$ = 3 nA and corresponding *m*, *n* and *h* progression.

The soma fires action potentials in response to injected current (*I*_*e*_) and excitatory synaptic input (*I*_*syn*_). The progression of the membrane potential is governed by the ion channel currents described. Figure 2 shows the somatic membrane potential over time in response to two current injections, and corresponding ion-channel parameters *m*, *n*, and *h*. A small, constant current injection can cause one somatic action potential to fire as the ion-channel parameters stabilize and adapt to the injected current (Figure 2A). A larger, constant current injection causes repeated firing of somatic action potentials and as the ion-channel parameters do not stabilize, firing is constant (Figure 2B). A LIF neuron is not able to adapt in this way and is either firing constantly or not firing at all.

#### 2.1.1. Numerical Methods

The neuron is modeled as an equipotential sphere such that the same electrical potential exists across the whole surface and hence the entire neuron can be described with a single membrane potential in a single compartment model. The ion-channels described in Section 2.1 (Equations 2–4) are incorporated into the general equation for a single compartment neuron in which the membrane voltage (*V*_*soma*_) is modeled over time. The progression of membrane voltage is calculated at discrete timesteps with interval Δ*t* = 0.1ms.


(8) $$\begin{array}{l} C_{m}\frac{dV_{soma}}{dt}=-I_{m}+\frac{I_{e}}{A} \end{array}$$

Membrane capacitance (*C*_*m*_) is uniformly set to 1 μFcm^2^ over the neuron. The conductance per unit area (*i*_*m*_) is defined in Equation (2) (S/cm^2^), $I_{e}^{\mu }$ is the total electrode current flowing into the compartment (nA) and *A* is the area of the neuron (mm^2^). Equation (8), combined with Equations (2), (3), and the corresponding equations for *m* and *h*, make up a system of ordinary differential equations (ODEs) where the rates of change of more than one variable are described: membrane voltage (*V*_*soma*_), sodium activation parameter (*m*), sodium inactivation parameter (*h*), and potassium activation parameter (*n*) (Figure 2).

### 2.2. Multi-Compartment Modeling

A two-compartment neuron morphology consisting of a somatic compartment and a dendritic compartment is designed incorporating ion-channel currents. Inspiration is drawn from the multi-compartment neuron model presented by Gidon et al. (2020) with the aim of simplifying this model in order to make it suitable for implementation on neuromorphic hardware while preserving the higher level L2/L3 pyramidal neural cell capabilities demonstrated. The dendritic compartment represents the apical dendrites and the somatic compartment represents the soma and basal dendrites (Figure 1B). For the somatic compartment, HH sodium and potassium ion-channels described in Section 2.1 are implemented. For the dendritic compartment, a calcium channel introduced by Gidon et al. (2020) is implemented in an attempt to capture the L2/L3 pyramidal neural cell firing dynamics demonstrated by the authors.

#### 2.2.1. Dendritic Currents

The dendritic compartment is modeled with the same leak current (*I*_*L*_) as the soma and a calcium current (*I*_*dCaAP*_) as described in Gidon et al. (2020). The calcium current in the dendritic compartment gives the compartment the ability to fire its own action potentials (independent of the somatic action potentials). These dendritic calcium action potentials are known as dCaAPs. The dCaAP current is activated when the dendritic membrane potential (*V*_*dend*_) crosses a threshold value (*V*_*thresh*_ = −36mV):


(9) $$\begin{array}{l} I_{dCaAP}=-\omega K\left(v\right)\left(A-B\right) \end{array}$$

with weight, ω = 3 (dimensionless). When *V*_*dend*_ crosses the threshold, *V*_*thresh*_, the dCaAP is activated: the activation function of the dendrite, *K*(*v*), is calculated and the time of dCaAP activation, *t*′, is set to the current timestep, *t*.


(10) $$\begin{array}{l} K\left(v\right)=\exp\left(\frac{-F\left(V_{dend}-V_{thresh}\right)}{\tau_{K}}\right) \end{array}$$

where *F* is a normalization factor *F* = 1/(*V*_*thresh*_ − *V*_*rest*_) and τ_*K*_ is the dCaAP amplitude decay constant τ_*K*_ = 0.3 (dimensionless). The dCaAP current has a 200ms refractory period in which it cannot fire.

*A* and *B* describe the rise and decay of the dCaAP current and are described by sigmoidal functions:


(11) $$\begin{array}{l} A=\frac{1}{1+\exp\left(-\frac{\left(t-t^{\prime }\right)}{\tau_{A}}\right)} \end{array}$$

(12) $$\begin{array}{l} B=\frac{1}{1+\exp(-\frac{\left(t-(t^{\prime }+\Delta t^{\prime }\right)}{\tau_{A}})} \end{array}$$

where *t*′ is the time of dCaAP activation, Δ*t*′ = 21ms, τ_*A*_ = 3ms and τ_*B*_ = 0.4ms.

The total membrane current in the dendritic compartment is calculated as the sum of the dCaAP current and the leak current:


(13) $$\begin{array}{l} I_{dend}=g_{L}\left(V_{dend}-E_{L}\right)+I_{dCaAP} \end{array}$$

**Figure 3:**
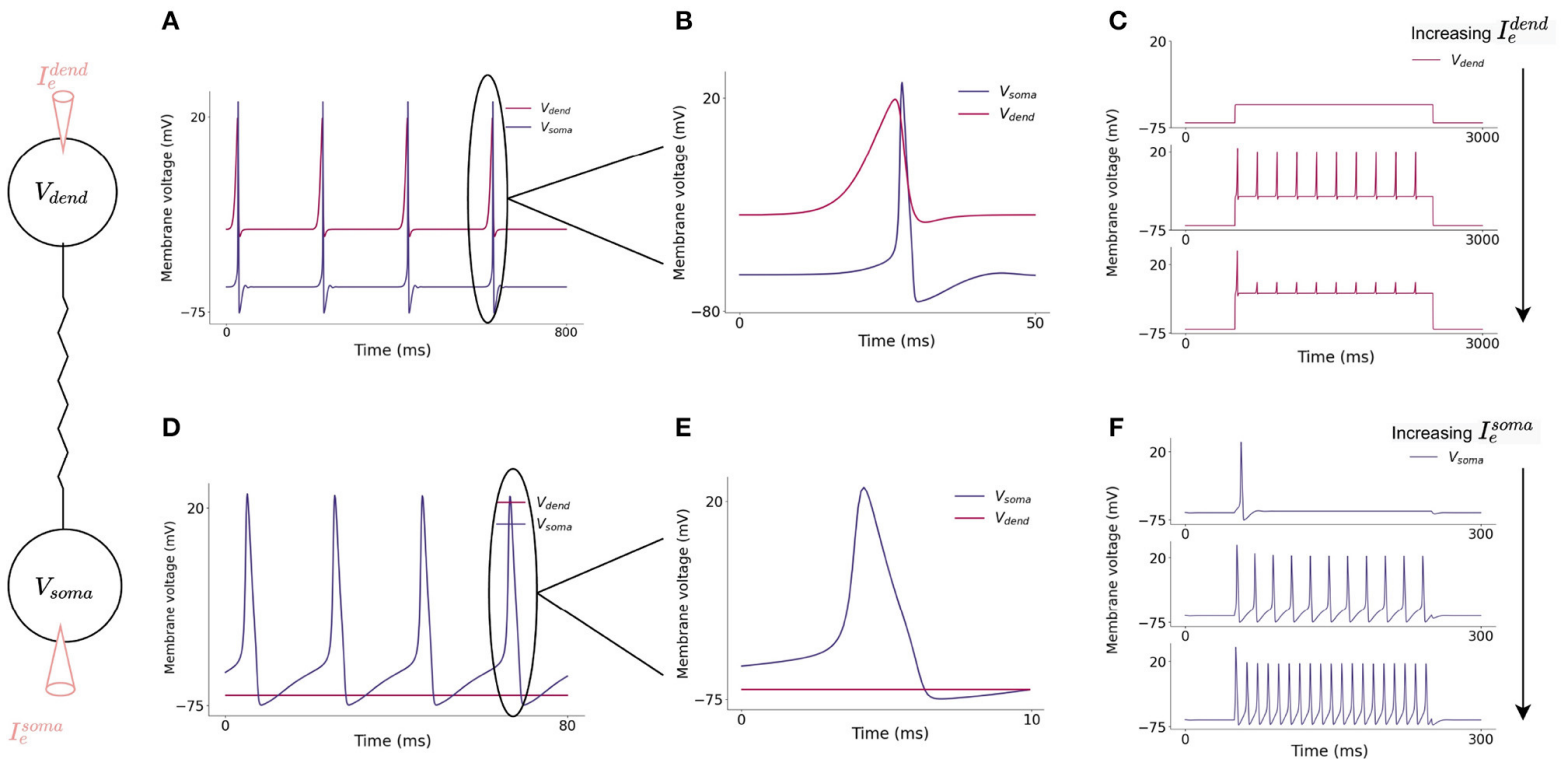
**(A)** Action potential initiation in dendritic and somatic compartment in response to a 3 nA injected current into the dendritic compartment. Dendritic compartment regularly fires dCaAPs with a refractory period of 200 ms. dCaAPs propagate to the somatic compartment and cause somatic action potentials. The dCaAP therefore precedes somatic action potentials **(B)**. **(C)** Spiking dynamics of the dendritic compartment in response to increasing current injections into the dendrite, the frequency of dendritic spikes remains constant but the amplitude decreases as the current injection increases. **(D)** Action potential initiation in somatic compartment in response to a 10 nA injected current into the somatic compartment, the dendritic compartment does not fire in response to this injected current **(E)**. **(F)** Spiking dynamics of the somatic compartment in response to increasing current injection into the soma, the frequency of somatic spikes increases as the current injection increases.

**Figure 4:**
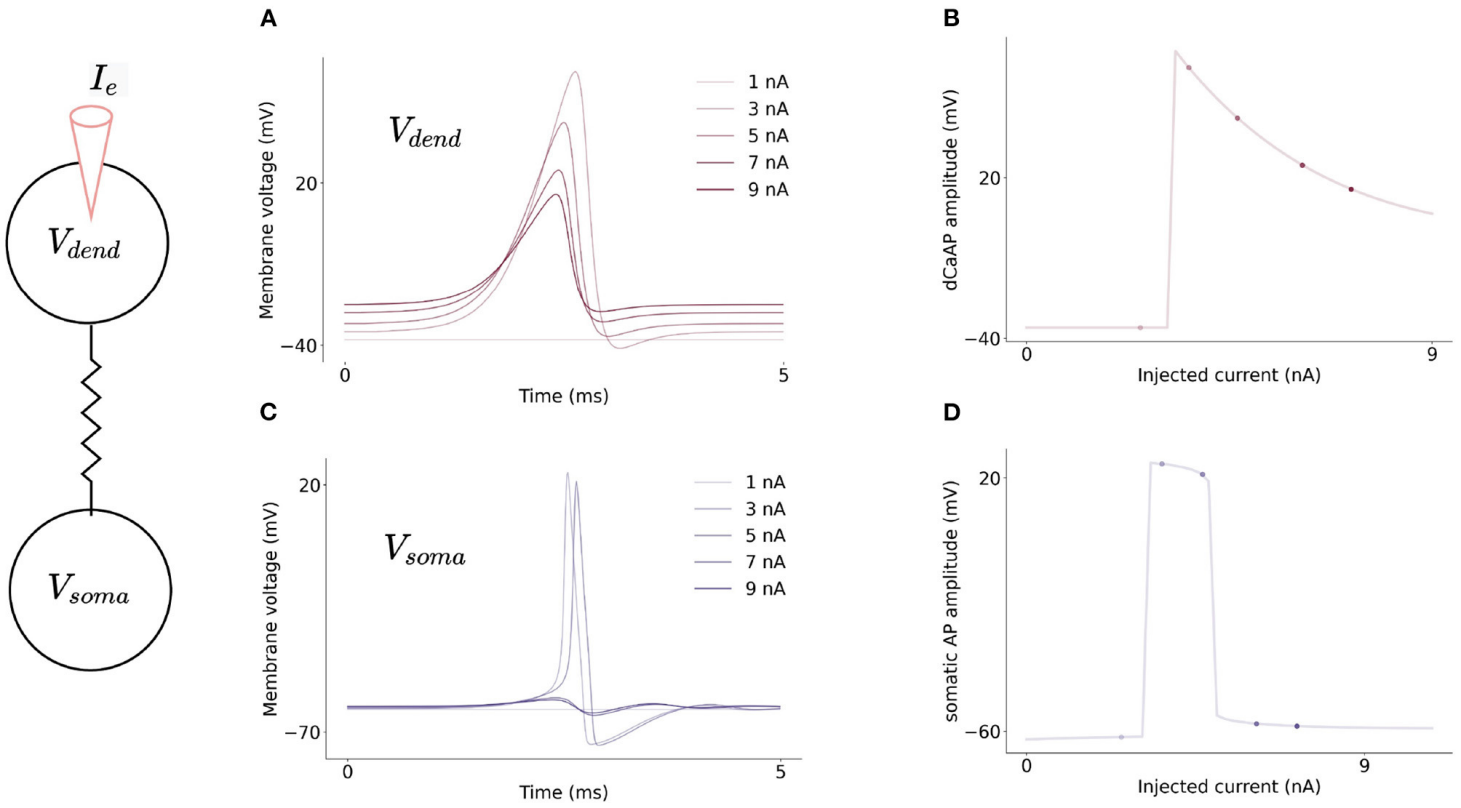
**(A)** Increasing injected current into the dendritic compartment results in a decrease in dCaAP amplitude. **(B)** The shape of the dCaAP is governed by the dendritic activation function, *K*(*v*) (Equation 10), which exhibits a characteristic shape in which the threshold current for dCaAP firing is the maximum dCaAP activation and hence the maximum dCaAP amplitude, the amplitude then decays with decay constant τ_*K*_ = 0.3 (dimensionless) (Equation 10). **(C)** Resulting somatic membrane voltage with increasing injected current into the dendritic compartment. Amplitude of somatic action potentials decreases with decreasing dCaAP amplitude. The all-or-nothing nature of these action potentials causes a lack of firing in the soma when the dendritic stimulus intensity gets higher. **(D)** Somatic action potential amplitudes are maximum when the dCaAP activation threshold is reached, they then decrease until the firing threshold for somatic action potentials is no longer reached and the soma stops firing.

Current flows from the dendritic compartment to the somatic compartment such that injected current into the dendrite can cause somatic action potentials to fire (Figure 3A) slightly after dendritic action potentials. Firing dynamics of the two compartment model in response to injected current into each compartment is presented in Figure 3. Increasing input to the dendritic compartment causes the amplitude of dCaAPs to decrease, this in turn causes somatic action potentials to stop firing as the current flowing to the somatic compartment will decrease with decreased amplitude of dCaAP (Figure 4).

#### 2.2.2. Numerical Methods

The single compartment model is described with a single membrane potential, however, membrane voltages actually vary considerably across the expansive surface of a neuron. It is possible to analyse signal propagation within neurons using a mathematical analysis known as “cable theory” (Dayan and Abbott, 2005). A two-compartment neuron is modeled using cable theory which assumes that the membrane potential varies with longitudinal distance along the axon, *x*, enabling it to be expressed as a partial differential equation (PDE) as a function of *x* and time, *t*, *V*(*x, t*):


(14) $$\begin{array}{l} c_{m}\frac{\partial V}{\partial t}=\frac{a}{2R}\frac{\partial^{2}V}{\partial x^{2}}-I_{m}+I_{e} \end{array}$$

where *R* is the intracellular resistivity (MΩmm^2^) and *a* is the radius (mm^2^). Appropriate boundary conditions are defined as the neuron is split into two compartments (soma and dendrite)—assuming membrane potential does not vary across the surface of the compartment—each with their own voltage (*V*_*soma*_ and *V*_*dend*_). This allows the continuous membrane potential, *V*(*x, t*), to be approximated by a set of membrane potential values in each compartment. Applying these boundary conditions simplifies the PDE to a system of ODEs for each compartment such that each compartment is described by its own membrane potential. For the somatic compartment, *V*_*soma*_:


(15) $$\begin{array}{l} c_{m}\frac{dV_{soma}}{dt}=-I_{m}^{soma}+\frac{I_{e}^{soma}}{A_{soma}}+g_{dend,soma}\left(V_{soma}-V_{dend}\right) \end{array}$$

For the dendritic compartment, *V*_*dend*_:


(16) $$\begin{array}{l} c_{m}\frac{dV_{dend}}{dt}=-I_{m}^{dend}+\frac{I_{e}^{dend}}{A_{dend}}+g_{soma,dend}\left(V_{dend}-V_{soma}\right) \end{array}$$

where $I_{e}^{soma}$ and $I_{e}^{dend}$ is the total electrode current flowing into the compartments (nA) and *A*_*soma*_ and *A*_*dend*_ is the area of the compartments (mm^2^). The constants *g*_*soma,dend*_ and *g*_*dend,soma*_ (nA/mm^2^) determine the resistive coupling between neighboring compartments. The membrane current for each compartment, $I_{m}^{soma}$ and $I_{m}^{dend}$ are described in Equations (2) and (9). At each timestep, the voltage update equation and corresponding activation parameters (Equations 15 and 16) must be solved, along with the corresponding activation parameters for any present ion channels such as Equation (3) for both compartments. A backwards Euler integration scheme is used due to its robust stability (Hines and Carnevale, 1997) by exploiting the conditional linearity of the ion-channel update equations (Dayan and Abbott, 2005).

### 2.3. Synaptic Model

Where synapses are incorporated into the model, synaptic currents are modeled as


(17) $$\begin{array}{l} I_{syn}=g_{syn}\left(V_{\mu }-E_{syn}\right) \end{array}$$

where *E*_*syn*_ is the reversal potential for the synaptic current (mV), and *g*_*syn*_ is the synaptic conductance (S/cm^2^). All synapses model excitatory NMDA connections, therefore *E*_*syn*_ = 0 mV (Dayan and Abbott, 2005). Synaptic conductance is modeled as:


(18) $$\begin{array}{l} g_{syn}=g_{max}*P_{s} \end{array}$$

where *g*_*max*_ = 0.05 is the maximal conductance and *P*_*s*_ is the probability of neurotransmitter release, modeled as:


(19) $$\begin{array}{l} P_{s}=P_{max}*\left(e^{\frac{-t}{\tau_{s}}}\right) \end{array}$$

where the maximal probability of neurotransmitter release *P*_*max*_ = 1 and τ_*s*_ = 10 ms. All synapses were activated at 20 Hz for simulations and are incorporated into dendritic (*I*_*dend*_, Equation 13) or somatic (*I*_*soma*_, Equation 2) currents as an additional term.

### 2.4. SpiNNaker Implementation

To make the models suitable for implementation on neuromorphic hardware, modifications to the system of equations are sought to decrease the computational load of simulation. Neuron models on SpiNNaker are written in C and compiled into ARM executable code. The SpiNNaker ARM968 CPU provides an energy-efficient core on which to simulate large-scale neural networks. This core has no floating-point hardware so fixed-point arithmetic is the preferred data representation. Two 32-bit fixed-point arithmetic types are used in this study which are defined in the ISO standard 18037 and are implemented by the GCC compiler. Variables and constants assuming values greater than 1 are defined as an ISO 10837 s16.15 *accum* fixed-point type: a signed 16-integer and 15-fractional bit number. Variables and constant taking values exclusively between 0 and 1 (for example *m*, *n*, and *h*) are defined as ISO 10837 u0.32 *unsigned long fract* fixed-point type: an unsigned 32-fractional bit number. Previous efforts to model more complex neuron models on SpiNNaker (Hopkins and Furber, 2015) reported spike time lag in comparison with reference models, however, later work (Hopkins et al., 2020) demonstrated that errors can be reduced by introducing various rounding techniques including *round-to-nearest* rounding. These methods are implemented here to reduce arithmetic error between the SpiNNaker implementation and the reference model. While most modern processors include hardware support for common arithmetic operations, SpiNNaker lacks hardware support for division and exponential operations. Simplifying assumptions which still give a biologically faithful model were sought enabling pre-calculation of operations such as divisions and exponentials. For example, in Equation (10), *F* and τ_*K*_ are constant to avoid the need to calculate a division at runtime. Lookup tables (LUTs) were also used to eliminate a number of costly calculations: Equations (3)–(7) are replaced by lookup operations.

#### 2.4.1. Ion-Channels and HH Neurons

For potassium and sodium ion-channels, LUTs (using 12 kB of memory) remove nine exponential and twelve division calculations involved in the calculation of gating variables for the ion-channels (Equation 3) which greatly improves the efficiency of the simulation (Table 1). Inspiration was taken from the NEURON simulation environment (Hines and Carnevale, 1997), in which LUTs are used as standard for Hodgkin-Huxley style ion channels. In NEURON, values of τ_*n*_, τ_*m*_, τ_*h*_, *n*_∞_, *m*_∞_, and *h*_∞_ are pre-calculated for values of *V*_*soma*_ at 1 mV intervals between values of −100 and +100 mV and the value of *V*_*soma*_ is used with interpolation to retrieve corresponding parameters from the table. Here, a similar table is tested in which instead of values of τ_*n*_, τ_*m*_, and τ_*h*_, values of exp(Δ*t*/τ_*n*_) (and similar for *m* and *h*) with Δ*t* = 0.1 were pre-calculated for each value of *V* to further decrease the amount of computation required at each timestep. While use of this table did decrease the computational requirements, the change from NEURON's standard LUT meant that discrepancies were introduced. To rectify this, an identical LUT to NEURON's was created, along with a LUT which stores values of exp(Δ*t*/τ_*n*_) with Δ*t* = 0.1 for values of τ_*n*_ (and similar for *m* and *h*). Therefore, instead of the complex equations required to solve Equation (3) and the similar equations for *m* and *h*, each state update then requires only three look-up operations per activation parameter followed by one addition and multiplication.

**Table 1:** Time taken to update the membrane voltage in models in μs on the SpiNNaker and Jib2 neuromorphic hardware in one 0.1ms timestep.

		**HH**		**Two comp**		**LIF**
		**No LUT**	**LUT**	**No LUT**	**LUT**	
SpiNNaker		99.6	8.34	153.67	12.91	0.32
Jib2	300 MHz	2.59	0.73	3.22	1.09	0.09
	150 MHz	5.19	1.45	6.45	2.18	0.19

*Values for the time taken to update neuron state in a Leaky-Integrate-and-Fire neuron is also presented for comparison (values from Rhodes et al., 2018)*.

#### 2.4.2. Two-Compartment Model

The same LUT described in Section 2.4.1 is used for the somatic compartment in the two-compartment model. For the dendritic compartment, another LUT (using 1.6kB of memory) is used in the calculation of the dCaAP current (*I*_*dCaAP*_) again improving efficiency. Here, *A* and *B* in *I*_*dCaAP*_ are each described by two divisions and an exponential operation which are particularly costly on SpiNNaker hardware. However, as the two terms are not themselves voltage dependent, calculating each term at every timestep is unnecessary; *A* and *B* have characteristic sigmoidal shapes which describe the rise and decay of the dCaAP current which can be pre-calculated and loaded onto the SpiNNaker chips such that the *A* − *B* calculation:-


(20) $$\begin{array}{l} \frac{1}{1+\exp(-\frac{\left(t-t^{\prime }\right)}{\tau_{A}})}-\frac{1}{1+\exp(-\frac{\left(t-(t^{\prime }+\Delta t^{\prime }\right)}{\tau_{A}})} \end{array}$$

is replaced by a single look-up operation.

## 3. Model Validation

To assess the accuracy of the proposed models on neuromorphic hardware, the models are benchmarked against the NEURON simulation environment in a number of simulations. Benchmarking involves comparison between the membrane potential on each platform at each timestep and comparison of the timing of spikes. Monitoring progression of membrane potential enabled a comparison of the numerical solvers on the different platforms and spike times give a broader comparison as spikes are the fundamental communication method in SNNs. Spike times are recorded as the timestep in which membrane voltage crossed a threshold value, −20 mV, and are compared between the different platforms. In order for direct comparisons to be made, identical simulations are run with SpiNNaker, Jib2 and with NEURON. Despite the mathematical complexity involved in these calculations, SpiNNaker and the Jib2 neuromorphic hardware are still able to model the HH and two-compartment neuron accurately.

### 3.1. Ion-Channels and HH Neurons

**Figure 5:**
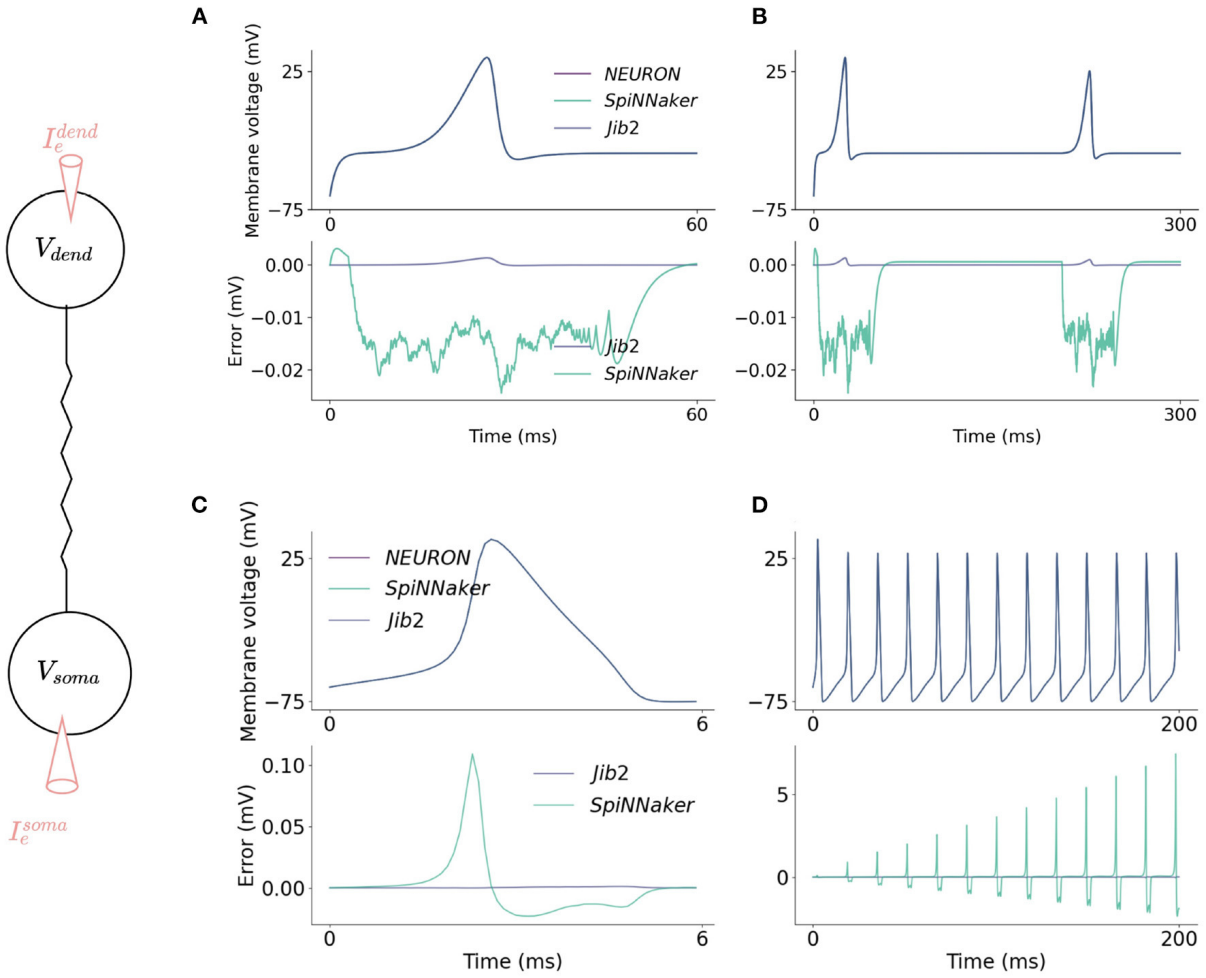
Accuracy comparisons between the two-compartment model implemented on SpiNNaker, Jib2 and NEURON. The dendritic compartment fires a dCaAP in response to a current injection of 3.5nA which is accurately modeled on the neuromorphic platforms **(A)**. The dendritic compartment is modeled accurately over time **(B)**: absolute errors are small throughout the duration of the action potential and drop to 0 mV when the dendritic compartment enters its refractory period. In the somatic compartment, absolute errors are larger because the calculation of *I*_*soma*_ is voltage dependent, therefore when errors are produced in this calculation, an error in voltage is calculated which then, in turn, further increases the error in the *I*_*soma*_ calculations **(C)**. Because of this, over time, SpiNNaker experiences accumulated errors. With Jib2 errors return to 0mV between spikes, and with do not accumulate over time **(D)**.

In the somatic model, current injections ranging from 0 to 10 nA are tested for 2 s of simulation time on both SpiNNaker and Jib2. This is long enough for steady state behavior to develop in the neuron and accumulated errors to become visible if present. The membrane voltage is then compared with an identical reference model in the NEURON simulation environment. The maximum error recorded over all current injections over full simulation time for SpiNNaker is 34.6mV, and for Jib2 is 0.106 mV. Spike times are consistent between Jib2 and NEURON, but the increase in error on the SpiNNaker neuromorphic system leads to accumulated errors which results in differences in spike timings between NEURON and SpiNNaker. Despite this, over the range of simulations, spike times on SpiNNaker only differ by one timestep (0.1 ms). In these neuron models the action potential is the most challenging part of the model due to the rapidly changing dynamics, and it is during action potentials that the largest errors between the fixed-point SpiNNaker implementation and the reference model are generated. One source of errors between these systems is the differing number representation: NEURON supports double precision floating point numbers, Jib2 supports single precision floating point units and SpiNNaker supports 32-bit fixed-point representations. During testing, switching the reference model to a CPU implementation and restricting the precision to 32-bit floating point arithmetic resulted in negligible errors between this implementation and Jib2. This shows that the different number representations are a source of error, these results are not included due to brevity. Another source of error in both SpiNNaker and Jib2 relative to NEURON is due to subtle differences in look-up tables being implemented: where NEURON pre-calculates values of τ_*n*_, τ_*m*_, and τ_*h*_ and then calculates values of exp(Δ*t*/τ_*n*_) (and similar for *m* and *h*), this exponential and division step is replaced with another LUT in the SpiNNaker implementation to avoid the need for exp(Δ*t*/τ_*n*_) calculations at each timestep. This source of error is confirmed by altering double precision reference models to mirror the SpiNNaker implementation and observing the decreased error. The accuracy of the somatic compartment is also tested with varying resolutions of LUT to further justify the use of a LUT with 1mV intervals between values of −100 and +100mV, as in NEURON (Hines and Carnevale, 1997) simulations. The model is simulated with no LUT, and with LUTs with 2 and 1mV voltage intervals. With no LUT, the maximum error was 105.9mV. Inclusion of LUTs increases the accuracy of models with the 2mV table resulting in a 59.2mV maximum error and the 1mV interval table providing the most accurate solution with a 34.6mV maximum error. SpiNNaker cores have 64kB of memory for data storage (DTCM). Finer resolution LUTs are not tested because they occupy more of the SpiNNaker DTCM and the accuracy of spike times using the 1mV interval is sufficient therefore occupying more DTCM with larger lookup tables is unnecessary. Despite the differing errors, both SpiNNaker and Jib2 can accurately model the HH model in response to a wide range of current injections and are able to maintain this accuracy over time (Figure 5).

### 3.2. Multi-Compartment Modeling

In the dendritic compartment, current injections ranging from 0 to 10nA are tested for 2 s of simulation time. Membrane voltage was recorded and absolute errors between SpiNNaker, Jib2 and NEURON are calculated, as well as the timings of spikes in both compartments. In the dendritic compartment, the maximum error recorded over all current injections over full simulation time for SpiNNaker is 0.00314mV, and for SpiNNaker 2 is 0.00137mV. An example current injection of 0.45nA into the dendritic compartment and resulting membrane potential recording is shown in Figure 5A. The evolution of absolute error over time in the dendritic compartment in response to injected current into the dendrite follows a typical shape each time a dCaAP is fired; the error remains below 0.5μV between spikes when the dendritic compartment is in its refractory period but rises as the membrane voltage rises, following a similar progression as the voltage. After the spike, the look-up table is no longer used and the value of *A* − *B* returns to 0, meaning the value of *I*_*dCaAP*_ is 0. There are therefore no issues with errors accumulating over time because after each spike the error returns to near zero as there is no active calcium current. The errors for the dendritic compartment simulations are smaller than the somatic model due to a decreased complexity in the equations (the soma contains more ion-channels). Again, errors result here from the differing numerical datatypes used on the different systems. This is demonstrated by restricting the reference model to single precision floats which decreases the error between Jib2 and the reference model. Again, during testing, switching the reference model to a CPU implementation and restricting the precision to 32-bit floating point arithmetic resulted in decreased errors between this implementation and Jib2, these results are not included due to brevity. The errors are not large enough to cause any differences in the timing of spikes between SpiNNake, Jib2 and the NEURON model. Spike times remained consistent across all platforms.

### 3.3. Performance Profiling

We are interested in accelerating brain simulation with neuromorphic hardware, therefore the time taken to update the state of a neuron in each simulated 0.1ms timestep is a key metric to evaluate for the different implementations. This is measured through recording the number of clock cycles taken to update the membrane potential in each model, and combining with the clock frequency. Each core on the SpiNNaker chip operates at 200MHz, meaning one clock cycle takes 5ns. With Jib2, voltage-frequency settings of 0.5V–150MHz and 0.8V–300MHz result in clock cycles taking 6.67 and 3.3ns, respectively. For each model, 100 neurons are profiled for 10,000 timesteps with a representative current injection causing regular somatic spiking with a frequency of 50Hz for HH and two compartment model. The amount of time taken to update the membrane potential in implemented models, with and without LUTs, is described in Table 1. For comparison, the time taken to update the membrane potential of a LIF neuron on SpiNNaker and Jib2 is also presented, the LIF neuron is kept sub-threshold during profiling in order to provide full state updates at each timestep, analogous to the HH and two compartment models.

In the HH model, the calculation of ion-channel parameters, resulting current values from these parameters, and subsequent membrane potential update for each 0.1ms timestep on SpiNNaker takes 99.6μs without any lookup tables (LUTs). Inclusion of the LUTs results in the update taking 8.34μs to compute, meaning the look-up table speeds the implementation up by over 11x. Similar calculations on Jib2 demonstrate the benefits of the hardware accelerators by showing a speed up in processing time. At 300MHz, the membrane potential update takes 0.73μs and at 150MHz, the update takes 1.45μs. Again, Jib2 illustrates that the LUTs improve the implementation speed, both the HH model and the two-compartment model are over 3x faster with the LUTs (Table 1). The HH model on Jib2 with LUTs is within an order of magnitude of the LIF neuron running on Jib2 which takes 0.09μs.

Addition of the calcium current in the dendritic compartment for the two-compartment model increases the amount of time taken to update the membrane potential. On SpiNNaker, updating the somatic membrane potential takes 12.91μs with LUTs, without LUTs this takes 153.67μs, over 11x longer. On Jib2 these membrane potential updates are quicker, taking 1.09 and 2.18μs with the core operating at 300 and 150MHz, respectively. Again, this model is 3x faster with the inclusion of the LUTs, with the non-LUT implementations taking 5.19 and 6.45μs (Table 1).

## 4. Results

### 4.1. HH Model Increases Expressiveness of Single Compartment Neurons

After models were validated (Section 3), the additional behaviors they bring to neuromorphic hardware were explored which have not been demonstrated previously on these platforms. The sodium and potassium ion-channels incorporated into a HH neuron model give the neuron a number of firing capabilities that are unable to be produced with simple LIF neurons. Izhikevich (2004) identified 20 of the most important neurocomputational spiking features of biological neurons which can be captured with spiking neuron models. The Hodgkin-Huxley model was identified to be able to reproduce all 20 of the firing dynamics, while the LIF neuron model can only reproduce 3: the ability to spike tonically, to increase firing frequency in response to increased input strength and the ability to integrate inputs and fire in response to them (Izhikevich, 2004). The single compartment neuron model here features Hodgkin-Huxley sodium and potassium ion-channels which therefore give this model the ability to produce all 20 neurocomputational features.

**Figure 6:**
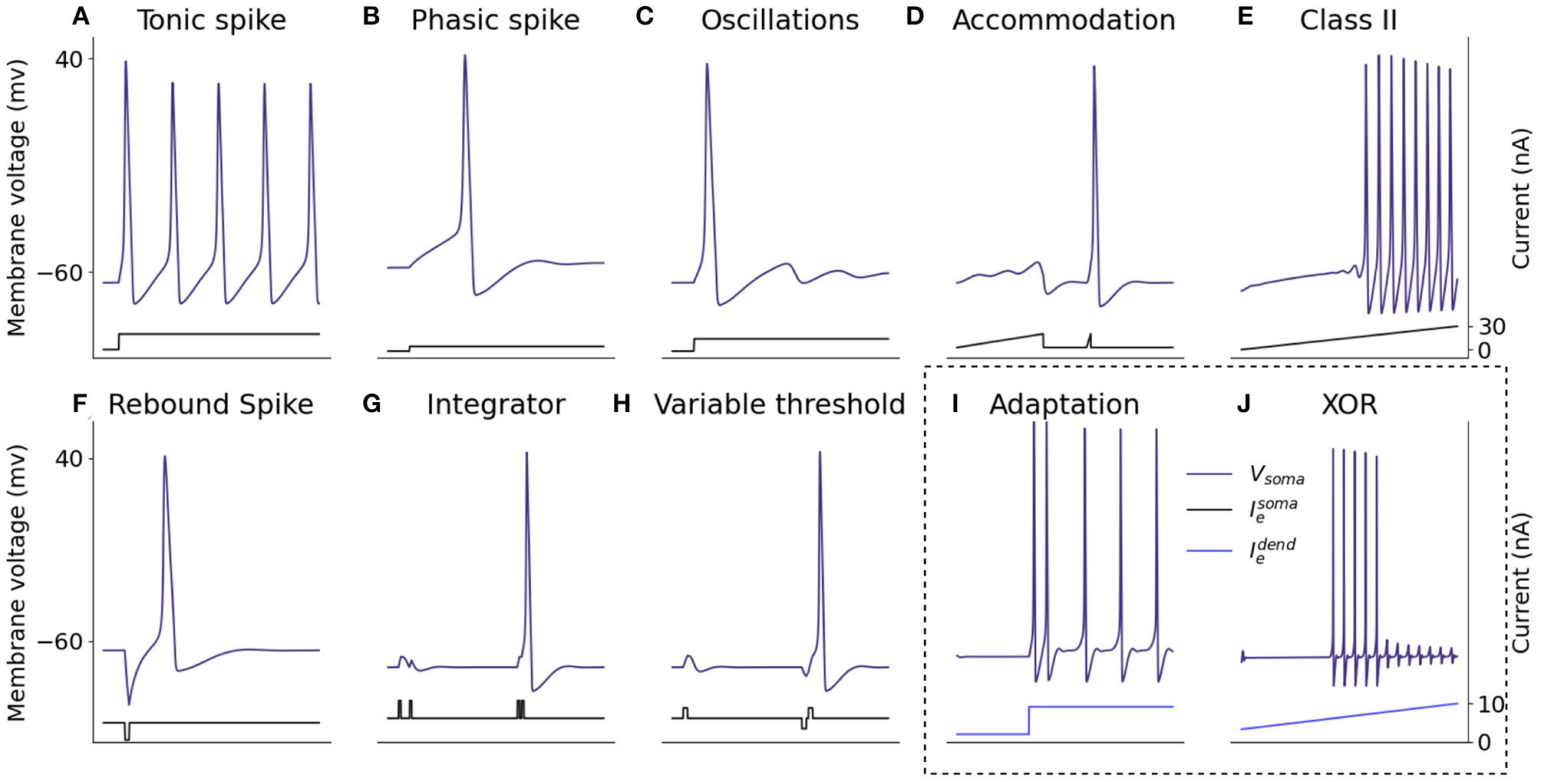
Spiking properties of the somatic compartment. Shown are simulations of the two-compartment model with current injected into the somatic or dendritic compartment to reproduce different firing dynamics capturing a number of biologically representative neural capabilities. The firing dynamics in the box are achieved through injected current into the dendritic compartment rather than the somatic compartment itself. **(A)** Tonic spike. **(B)** Phasic spike. **(C)** Oscillations. **(D)** Accommodation. **(E)** Class II. **(F)** Rebound spike. **(G)** Integrator. **(H)** Variable threshold. **(I)** Adaptation. **(J)** XOR.

Firing features of the somatic model are demonstrated in Figure 6, through injection of current directly into the neuron and recording the resulting somatic membrane voltage. It is not possible for a single neuron model to exhibit all properties simultaneously because some features, for example the ability to fire a train of spikes in response to a constant input, and the ability to fire periodic bursts of spikes in response to constant input, are mutually exclusive. For that reason, 10 biologically important firing features are presented that can be exhibited simultaneously without altering parameters from those described in Section 2.2. In response to a constant somatic current injection, the soma can fire a constant train of spikes known as tonic spiking (Figure 6A). If the current injection is just strong enough to cause a spike, the neuron demonstrates phasic spiking (Figure 6B) where a single spike is fired followed by inactivity. Phasic spikes are often followed by sub-threshold oscillations (Figure 6C) caused by ion-channel currents. Inputs to neurons are generally not constant, and neurons can display a number of firing properties in response to different input currents. Neurons can demonstrate accommodation to inputs: presenting the neuron with a slowly increasing current does not produce a spike but presenting the same neuron with a sharply increasing current will produce a spike (Figure 6D) due to the ion-channels within the neuron having more time to adapt to the current, meaning the neuron accommodates. Hodgkin-Huxley neurons are Class II excitable neurons meaning they are either inactive or they fire spikes with a high frequency, this is displayed by presenting the neuron with a steady increase in injected current (Figure 6E). Adaptation of ion-channel currents also leads to a phenomenon in which the neuron fires a spike after an inhibitory current injection (Figure 6F) known as a post-inhibitory spike. Neuron models in SNNs generally integrate spiking inputs over time, if inputs are closer together then the neuron is more likely to fire spikes as the firing threshold is passed (Figure 6G). While most SNN neuron models have fixed voltage threshold for firing spikes, biological neurons actually have a variable threshold which is determined by the activity of the neuron (Figure 6H). Briefly exciting the neuron in Figure 6H is not enough to make the neuron fire, however if it is preceded by a brief inhibitory input, this same excitation will cause the neuron to fire. Spike rate adaptation is a phenomenon in which neurons fire tonic spikes with decreasing frequency, this feature is mutually exclusive with the tonic spiking ability discussed above and single-compartment neuron models are unable to display both properties. Here, inclusion of the dendritic compartment allows the soma to display spike-rate adaptation in response to a constant current injected into the dendritic compartment (Figure 6I). Injecting the dendritic compartment with a steady increase in injected current leads to remarkably different somatic firing dynamics in which firing starts when input is above threshold but ceases firing when the input continues to rise (Figure 6J), this phenomenon is explained in Section 4.2.

Other neurocomputational properties presented by Izhikevich can be captured by altering the parameters described in Section 2.2. These include the ability to burst (rather than tonically) fire and to fire in response to inhibitory (rather than excitatory) inputs. Properties such as these can be captured by altering parameters involved in the differential equations describing ion-channel currents (Kirigeeganage et al., 2019). Sodium channel currents change more rapidly than potassium currents in the beginning of the progression of an action potential, they are described by an activation variable (*m*) and an inactivation variable (*n*) (Section 2.1). Therefore, to adjust the neuron to be responsive to inhibitory inputs, modifications to the differential equation describing *m* can be made to alter the responsiveness of the neuron.

Izhikevich compared 11 spiking neuron models by the ability of the models to produce some of these features and the computational cost of each model (Izhikevich, 2004). The Hodgkin-Huxley model was the only one able to produce all firing properties while also being biophysically meaningful. This biological accuracy leads to higher computational cost of the model which makes it more expensive to implement than other neuron models. However, computational costs can be diminished using a variety of techniques (see Section 2.4). In addition, the biophysical plausibility of the Hodgkin-Huxley model allows incorporation of dendritic morphology and different ion-channels through cable equation modeling, this is not possible with less biologically plausible models. The dendritic modeling in the second compartment gives the neuron additional computational properties to further increase the firing capabilities beyond those identified by Izhikevich, described in Section 4.2.

### 4.2. Dendritic Compartment Enables Single Neuron to Function as a Multi-Layer Network

Inclusion of the dendritic compartment further increases the computational properties of the neuron beyond the 20 identified by Izhikevich. The dynamics described by Izhikevich are relatively well-known capabilities for Hodgkin-Huxley neurons and can be reproduced by other neuron models including the model proposed by Izhikevich himself (Izhikevich, 2004). However, the biological plausibility of the Hodgkin-Huxley model enables it to be built upon through the incorporation of more compartments representing dendritic branches which further increase the capabilities of the neuron.

Here, the dendritic compartment gives the neuron the ability to compute a logical operation known as exclusive-or (XOR). Logical operations are performed on binary inputs and produce a binary output. An XOR operation is a logical operation in which an exclusive-or is implemented: the binary output is 1 (or true) when there is only one input to the operation, if both of the inputs are 0 (or false) or both of the inputs are 1 then the output of the XOR operation is 0 (Table 2). While simple logic operations such as AND and OR are easily implemented in single units within neural networks, the XOR function is a common problem in neural network research and is widely used as an example of a linearly inseparable problem; it has become a benchmark in machine learning for testing neural network capabilities in solving complex problems. SNN implementation of the XOR operation has thus far required multiple layers of spiking neurons as the nature of spiking neural network architectures is that each layer can only separate data points with a single line (Vogels and Abbott, 2005; Reljan-Delaney and Wall, 2018; Cyr et al., 2020). XOR functions were deemed impossible in single-layer networks—Marvin Minsky and Seymour Papert provided proof that single-layer ANNs could not perform XOR in their 1969 book Perceptrons (Minsky and Papert, 2017) due to the non-linear separability. An XOR gate was demonstrated within a large SNN by (Vogels and Abbott, 2005) who stated that “a functional XOR gate requires ~220 neurons”. Here, the XOR problem is solved with a single neuron model.

**Table 2:** XOR truth table demonstrating the binary output of an XOR operation in response to two binary inputs.

**A**	**B**	**A XOR B**
0	0	0
0	1	1
1	0	1
1	1	0

*If the inputs are both 1 (true) or both 0 (false) then the output is 0 (false), if either input is 1 and the other 0 then the output is 1*.

**Figure 7:**
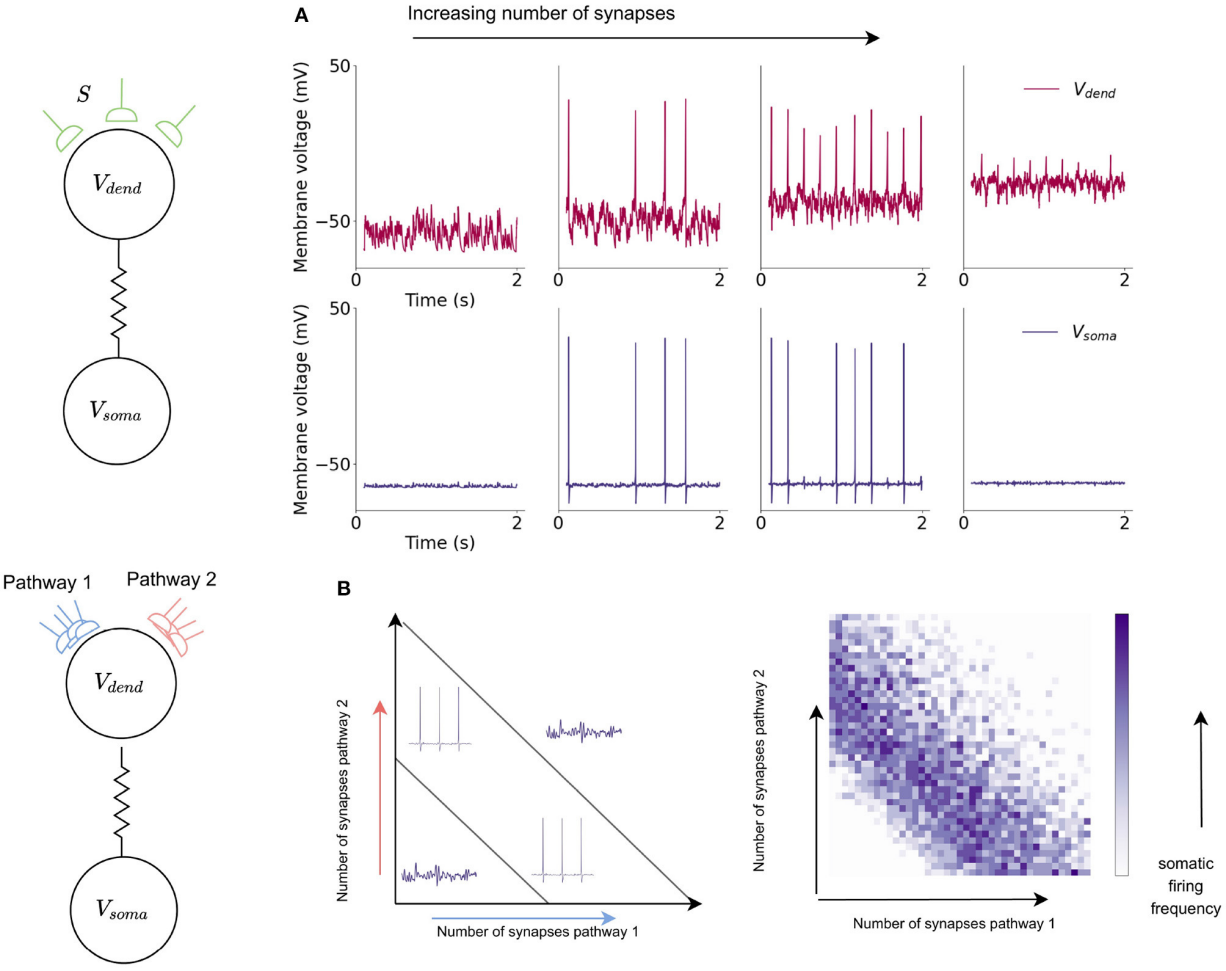
Somatic compartment exhibits XOR response to dendritic input in a single neuron model. **(A)** Dendritic and somatic firing dynamics in response to increased synaptic input into the dendritic compartment. Increased number of synapses leads to an initial increase and then decrease in dCaAP amplitude which subsequently cause the somatic compartment to start firing action potentials then stop. **(B)** The dynamics of the somatic compartment in response to the dendritic inputs provide a solution to the XOR problem in a single neuron model. Somatic compartment exhibits XOR response to dendritic input in a single neuron model. Increased number of synapses leads to an initial increase and then decrease in dCaAP amplitude which subsequently cause a similar increase and cease of somatic action potential firing.

The shape of the dendritic activation function allows the XOR problem to be solved here with a single neuron model. The activation function results in the amplitude of dCaAPs decreasing when the input to the dendritic compartment increases above a certain strength; the dCaAP amplitude is maximal when the input to the dendrite crosses the threshold for activation, then decreases as the input increases further (Figure 7A). As the dCaAP amplitude decreases, the amount of current flowing from the dendritic compartment to the somatic compartment decreases which in turn decreases the somatic action potential amplitude (Figure 4B). As somatic action potentials are an all-or-nothing spike response, when the current flowing from the dendritic compartment to the somatic compartment decreases below a certain value, the soma stops firing action potentials (Figure 4B). Therefore, the somatic compartment will start firing when the input to the dendrite is increased to its firing threshold and then will decrease and eventually stop firing as input is increased further. Similar behavior is observed when input to the dendritic compartment is synaptic rather than injected current. Increasing the number of synapses also causes the dCaAP amplitude to increase then to decrease above a certain number of synapses, leading to somatic action potential firing and subsequent cease (Figure 7).

While the action potentials arising from calcium currents in the dendritic compartment are responsible for XOR-type computation, the somatic compartment, through integration of sodium and potassium currents, computes standard logical operations for spiking neurons such as AND and OR. The combination of these differing logical operations allow the neuron to act as a multi-layer network, increasing the computational capabilities of a single neuron model in comparison with a leaky integrate-and-fire model.

## 5. Discussion

This work has provided the first fixed-point implementation of ion-channel, Hodgkin-Huxley, and multi-compartment models on SpiNNaker neuromorphic hardware and the first profiling for both speed and accuracy of such models on SpiNNaker2 prototype neuromorphic hardware, demonstrating the improved performance of the next-generation system through the use of hardware accelerators and floating point arithmetic. The first demonstration of a two-compartment neuron model running on neuromorphic hardware that can solve the XOR problem using a single neuron is also presented through this work.

Neuromorphic systems are designed to provide low-energy platforms for simulation of Spiking Neural Networks (SNNs) but in doing so biologically plausible neuron models have largely been ignored in favor of simple and efficient neuron models such as the Leaky Integrate-and-Fire (LIF) model. In contrast, focus in the computational neuroscience community has been on building models with a high degree of biological accuracy which are in turn accompanied by large computational costs, making the models difficult to scale into SNNs. This work bridges this gap by presenting two biologically inspired neuron models (Figure 1), implemented efficiently and accurately on SpiNNaker and Jib2 neuromorphic platforms (Figure 5): a single compartment Hodgkin-Huxley (HH) neuron (Figure 2) and a multi-compartment neuron incorporating dendritic computation (Figure 4).

Both SpiNNaker and Jib2 are able to accurately model both neurons over time with identical spike times recorded on Jib2 and a reference model in NEURON (Hines and Carnevale, 1997) and spike times within 0.1ms on SpiNNaker. Manipulation of equations, pre-calculation of constants and the use of lookup tables (using 12 and 13.6kB of memory for HH and two-compartment models, respectively) enabled a significant speed up of simulation time of the models (approx 11× for both the single and two-compartment models—Table 1). This speed-up is further increased by 3× with implementation on the next-generation Jib2 neuromorphic chip, demonstrating the effectiveness of hardware accelerators for expressions such as exponential operations (Table 1) when simulating biologically representative neurons.

Comparison with neuromorphic implementations of the conventional LIF neuron model revealed that both the HH and the multi-compartment neurons were slower to simulate on neuromorphic hardware, due to the increased complexity of the models (Table 1). However, the computational capabilities gained justify the increased expense of running the model, and the model on Jib2 is within an order of magnitude of the LIF neuron in terms of computation time. The underlying ion channel models directly correspond to biophysiological data, bringing increased biological relevance to models simulated on neuromorphic hardware. Furthermore, the presented HH model exhibits a wide range of firing characteristics which cannot be captured with LIF neurons (Figure 6), and the inclusion of a dendritic compartment enables the a single neuron model to function as a multi-layer network. The multi-compartment model provides the first implementation of a single neuron model capable of solving the XOR problem on neuromorphic hardware (Figure 7).

This work has explored simulation and profiling of individual neurons, and their realization on neuromorphic systems. The ultimate goal of implementing these models is harnessing their ability to capture biologically representative features in *large-scale* SNNs, and opening up new applications in bio-inspired AI. To understand how the presented models would scale when included in large networks, it is useful to contrast performance with LIF neurons and biologically representative neural circuits previously evaluated on SpiNNaker. In previous work modeling cortical microcircuits comprising LIF neurons, it was shown that neuron and synapse processing could be parallelized effectively on multicore architectures such as SpiNNaker (Rhodes et al., 2020). Through this parallelization real-time simulation of cortical circuits containing 80k neurons and 300M synapses was demonstrated, with an energy per synaptic event of ≈ 0.6 μJ. The models presented here would impact the *neuron* processing, resulting in a ≈ 40× reduction in neuron density relative to LIF neurons to accommodate the increased model complexity. This indicates that approximately 40× more SpiNNaker chips would be required to simulate the same size of model, leading to the same factor increase in total energy consumption. Projecting these numbers on to SpiNNaker2 requires consideration of the updated performance achieved with the new hardware. The HH and two-compartment neurons occupy 48 and 55kB, respectively (of the 128kB fast-access SRAM for combined instruction and data storage on Jib2) with the instructions to update the neuron and the storage of constants, variables and LUTs. Increasing the number of neurons does not significantly increase the storage requirements, as the instructions for updating the neurons are the same and all neurons share common LUTs. While the number of neurons per core determines the amount of state variables to be stored, these datastructures are relatively small compared to those described above (assuming split neuron and synapse processing/storage as described above, Peres and Rhodes, 2022). Therefore the determining factor in the number of neurons which can be simulated on each core is the processing time. As it takes 0.73 μs to update a HH neuron and 1.09 μs for the two-compartment neuron using a 0.1 ms simulation timestep, assuming the goal of real-time simulation, an upper limit of 136 HH neurons or 91 two-compartment neurons could be updated by a single core while maintaining real-time execution. In reality this number is likely to be reduced to enable cores to perform auxilliary operations such as monitoring and data recording, reducing overall neuron density. However, this is likely to remain above the 64 neurons per core utilized in previous cortical simulations on SpiNNaker (Rhodes et al., 2020), enabling real-time cortical simulations containing biologically representative ion-channel-based neuron models (on SpiNNaker2). Furthermore, embedding these models within the SpiNNaker routing and communications fabric should facilitate further expansion of model sizes while maintaining real-time execution. This indicates that the cost of changing from LIF to multicompartment models on SpiNNaker2 will incur a 10× increase in energy, with the overall system significantly more energy efficient—LIF neurons have been profiled at 20pJ per synaptic event (Höppner et al., 2021).

The model provides a framework for capturing and testing more biologically plausible neural dynamics in an efficient way. For example, different ion-channels can easily be substituted or added to the model, and more complex morphologies can be captured through inclusion of more dendritic compartments. Recent work has demonstrated the potential of multi-compartment neuron models to learn *via* a synaptic learning rule (Bicknell and Häusser, 2021), opening the door to the possibility of training the neuron models presented in this work within large-scale SNNs on neuromorphic hardware, in particular those featuring hardware accelerators to maximize efficiency. Significant computational capabilities are gained with each individual neuron model and neuromorphic architectures can provide energy-efficient platforms for simulations. While this work has focused on demonstrating feasibility through development of software models suitable for execution on SpiNNaker, the developed models also provide the first step toward algorithm-hardware co-design. Hardware requirements such as arithmetic operations and memory use have been identified, providing insights into how future neuromorphic systems could be tailored to further optimize execution.

## Data Availability Statement

The raw data supporting the conclusions of this article will be made available by the authors, without undue reservation.

## Author Contributions

MW designed and completed the project through model creation, implementation, validation, timing measurements, accuracy measurements and testing of models on both neuromorphic systems and NEURON, and wrote the manuscript. OR provided input into model design and implementation, assisted with efficiency improvements and measurements of models on both neuromorphic systems. Both authors reviewed and edited manuscript, and approved the submitted version.

## Funding

This work was supported by the EU ICT Flagship Human Brain Project (H2020 785907 and 945539).

## Conflict of Interest

The authors declare that the research was conducted in the absence of any commercial or financial relationships that could be construed as a potential conflict of interest.

## Publisher's Note

All claims expressed in this article are solely those of the authors and do not necessarily represent those of their affiliated organizations, or those of the publisher, the editors and the reviewers. Any product that may be evaluated in this article, or claim that may be made by its manufacturer, is not guaranteed or endorsed by the publisher.

## Footnotes

^1^All models discussed in the text are available at https://github.com/mollie-ward/beyondLIFNeurons, and can be compiled and run on any GCC compatible platform.
